# The genetic association study between polymorphisms in uncoupling protein 2 and uncoupling protein 3 and metabolic data in dogs

**DOI:** 10.1186/1756-0500-7-904

**Published:** 2014-12-11

**Authors:** Chihiro Udagawa, Naomi Tada, Junzo Asano, Katsumi Ishioka, Kazuhiko Ochiai, Makoto Bonkobara, Shuichi Tsuchida, Toshinori Omi

**Affiliations:** Department of Basic Science, School of Veterinary Nursing and Technology, Faculty of Veterinary Science, Nippon Veterinary and Life Science University, 1-7-1 Kyonan-cho, Musashino, Tokyo, 180-8602 Japan; Department of Veterinary Nursing, School of Veterinary Nursing and Technology, Faculty of Veterinary Science, Nippon Veterinary and Life Science University, 1-7-1 Kyonan-cho, Musashino, Tokyo, 180-8602 Japan; Department of Veterinary Clinical Pathology, Nippon Veterinary and Life Science University, 1-7-1 Kyonan-cho, Musashino, Tokyo, 180-8602 Japan; Laboratory of Comparative Cellular Biology, Nippon Veterinary and Life Science University, 1-7-1 Kyonan-cho, Musashino, Tokyo, 180-8602 Japan

**Keywords:** Dog, SNP, Indel polymorphism, UCP2, UCP3, Association study

## Abstract

**Background:**

The uncoupling proteins (UCPs) in the mitochondrial inner membrane are members of the mitochondrial anion carrier protein family that play an important role in energy homeostasis. Genetic association studies have shown that human *UCP2* and *UCP3* variants (SNPs and indels) are associated with obesity, insulin resistance, type 2 diabetes mellitus, and metabolic syndrome. The aim of this study was to examine the genetic association between polymorphisms in *UCP2* and *UCP3* and metabolic data in dogs.

**Results:**

We identified 10 SNPs (9 intronic and 1 exonic) and 4 indels (intronic) in *UCP2*, and 13 SNPs (11 intronic and 2 exonic) and one indel (exonic) in *UCP3*, by DNA sequence analysis of 11 different dog breeds (n = 119). An association study between these *UCP2* and *UCP3* variants and the biochemical parameters of glucose, total cholesterol, lactate dehydrogenase and triglyceride in Labrador Retrievers (n = 50) showed that none of the *UCP2* polymorphisms were significantly associated with the levels of these parameters. However, four *UCP3* SNPs (intron 1) were significantly associated with total cholesterol levels. In addition, the allele frequencies of two of the four SNPs associated with higher total cholesterol levels in a breed that is susceptible to hypercholesterolemia (Shetland Sheepdogs, n = 30), compared with the control breed (Shiba, n = 30).

**Conclusion:**

The results obtained from a limited number of individuals suggest that the *UCP3* gene in dogs may be associated with total cholesterol levels. The examination of larger sample sizes and further analysis will lead to increased precision of these results.

**Electronic supplementary material:**

The online version of this article (doi:10.1186/1756-0500-7-904) contains supplementary material, which is available to authorized users.

## Background

The uncoupling proteins (UCPs) in the mitochondrial inner membrane are members of the mitochondrial anion carrier protein family [1, 2]. Mammals have five UCP homologs, of which UCP1, UCP2, and UCP3 are closely related, while UCP4 and UCP5 are more divergent from the other UCPs [3].

Based on genetic association studies, *UCP2*, *UCP3*, or both are reportedly associated with obesity, insulin resistance, type 2 diabetes mellitus, and metabolic syndrome in humans [4–11]. For example, a SNP in the 5′ untranslated region in human *UCP3*, the UCP3 -55CT SNP, is known to be a genetic marker associated with mRNA expression [12], elevated high density lipoprotein cholesterol levels, a reduced body mass index (BMI), weight, waist circumference, waist to hip ratio, fat mass, low density lipoprotein (LDL) cholesterol, and total cholesterol (T-Cho) [13–15].

The treatment and prevention of obesity and metabolic-related diseases are also clinically important in dogs [16–25]. Our previous report showed that the nucleotide sequences, predicted amino acid sequences and the genomic structures of human *UCP2* and *UCP3* are highly homologous to the canine orthologs [26, 27]. In this study, we investigate whether the dog *UCP2* and *UCP3* genes are associated with alterations in metabolism.

## Results and discussion

**Figure 1:**
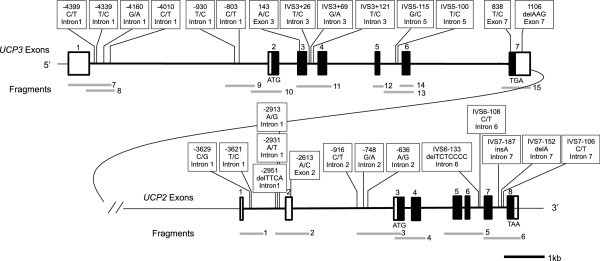
**Schematic representation of the DNA polymorphisms detected in the** ***UCP2*** **and** ***UCP3*** **genes in dog.** □: Exon(UTR) ■: Exon(CDS) ▬: Intron │: SNP or INDEL : PCR Fragment. The position of identified DNA polymorphism was numbered from the A of the initiator methionine ATG codon as the +1 revealed in exon. In case of intron, a positive number indicates the number of nucleotides away from the previous exon, while a negative number indicates the number of nucleotide away from the next exon.

Figure 1 shows a schematic representation of the canine *UCP2* and *UCP3* genes and the identified DNA polymorphisms from 119 animals from 11 breeds. For analysis of the dog *UCP2* gene, six regions were individually amplified from genomic DNA and sequenced. We then identified 10 SNPs (9 intronic and 1 exonic) and 4 indels (intronic) in *UCP2* (Figure 1, Additional file 1). In the dog *UCP3* gene, 13 SNPs (11 intronic and 2 exonic) and 1 indel (exonic) were revealed by sequencing nine regions of this gene (Figure 1, Additional file 1).

To test the association between the dog *UCP2* and *UCP3* genes and metabolic data, we determined the genotype of 50 Labrador Retrievers for each of 14 polymorphic sites (10 SNPs and 4 indels) in the *UCP2* gene, and examined whether any of the genotypes were associated with biochemical measurements of glucose (GLU), total cholesterol (T-Cho), lactate dehydrogenase (LDH), or triglyceride (TG). To exclude any contamination by disease of the animals, we selected Labrador Retrievers that had undergone a health examination for breeding for guide dogs by the Kyushu Guide Dog Association.

The average of measurements was calculated with respect to the genotype group. Nine of the 14 loci in the *UCP2* gene were polymorphic in this population of Labrador Retrievers. None of these DNA polymorphisms in the *UCP2* gene were significantly associated with any of the biochemical parameters in this study (Additional file 2). We also subjected the 14 polymorphic sites (13 SNPs and 1 indel) in the *UCP3* gene to this association analysis. Ten of the 14 sites were polymorphic in this population of Labrador Retrievers. There were no significant differences between genotype and GLU, LDH, or TG measurements for any polymorphic site. However, the T-Cho levels differed significantly among the genotype groups at four sites: -4399C/T, -4339T/C, -930T/C and -803C/T in intron 1 of the *UCP3* gene (*UCP 3* intron1). The average T-Cho levels in dogs carrying CC or CT at -4399 C/T were 273.5 ± 49.0 and 237.2 ± 53.3, respectively. The average T-Cho levels for the TT, TC, or CC genotypes at -4339T/C and -930T/C were 264.3 ± 49.6, 276.9 ± 49.5, and 233.5 ± 51.2, respectively. Those for CC or CT at -803C/T were 271.6 ± 49.5 and 239.1 ± 54.5, respectively (Table 1). The genotype distributions were in a Hardy–Weinberg equilibrium.

**Table 1:** **Association analysis of** ***UCP3*** **DNA polymorphisms with biochemical parameters among healthy Labrador Retrievers**

DNA polymorphism	Genotype	GLU	T-Cho	LDH	TG
*UCP3*	CC (34)	97.1 ± 8.4	273.5 ± 49.0	55.9 ± 18.0	44.8 ± 21.1
-4399C/T	CT (16)	98.8 ± 14.5	237.2 ± 53.3	55.7 ± 13.5	49.4 ± 24.8
	TT (0)	-	-	-	-
	CC vs CT + TT	0.597	**0.021** *	0.965	0.504
*UCP3*	TT (8)	94.5 ± 5.4	264.3 ± 49.6	55.3 ± 13.7	40.0 ± 13.5
-4339T/C	TC (27)	96.3 ± 11.5	276.9 ± 49.5	58.2 ± 20.9	50.4 ± 24.8
	CC (15)	101.7 ± 10.4	233.5 ± 51.2	51.9 ± 5.3	42.3 ± 20.5
	TT vs TC + CC	0.366	0.890	0.914	0.388
	TT + TC vs CC	0.079	0.011*	0.279	0.408
*UCP3*	CC (15)	94.4 ± 11.9	255.5 ± 52.6	56.9 ± 16.1	51.2 ± 23.2
-4010C/T	CT (29)	98.7 ± 10.2	270.2 ± 52.2	56.5 ± 18.5	43.2 ± 21.5
	TT (6)	100.7 ± 8.6	237.7 ± 54.7	50.0 ± 0.0	48.7 ± 24.6
	CC vs CT + TT	0.159	0.580	0.763	0.310
	CC + CT vs TT	0.462	0.234	0.362	0.782
*UCP3*	TT (8)	94.5 ± 5.4	264.3 ± 49.6	55.3 ± 13.7	40.0 ± 13.5
-930T/C	TC (27)	96.3 ± 11.5	276.9 ± 49.5	58.2 ± 20.9	50.4 ± 24.8
	CC (15)	101.7 ± 10.4	233.5 ± 51.2	51.9 ± 5.3	42.3 ± 20.5
	TT vs TC + CC	0.366	0.890	0.914	0.388
	TT + TC vs CC	0.079	0.011*	0.279	0.408
*UCP3*	CC (35)	96.9 ± 8.4	271.6 ± 49.5	55.7 ± 17.7	44.5 ± 20.9
-803C/T	CT (15)	99.5 ± 14.8	239.1 ± 54.5	56.1 ± 13.9	50.5 ± 25.2
	TT (0)	-	-	-	-
	CC vs CT + TT	0.431	0.045*	0.950	0.388
*UCP3*	TT (15)	94.4 ± 11.9	255.5 ± 52.6	56.9 ± 16.1	51.2 ± 23.2
IVS3+26T/C	TC (29)	98.7 ± 10.2	270.2 ± 52.2	56.5 ± 18.5	43.2 ± 21.5
	CC (6)	100.7 ± 8.6	237.7 ± 54.7	50.0 ± 0.0	48.7 ± 24.6
	TT vs TC + CC	0.159	0.580	0.763	0.310
	TT + TC vs CC	0.462	0.234	0.362	0.782
*UCP3*	GG (15)	94.4 ± 11.9	255.5 ± 52.6	56.9 ± 16.1	51.2 ± 23.2
IVS3+69G/A	GA (29)	98.7 ± 10.2	270.2 ± 52.2	56.5 ± 18.5	43.2 ± 21.5
	AA (6)	100.7 ± 8.6	237.7 ± 54.7	50.0 ± 0.0	48.7 ± 24.6
	GG vs GA + AA	0.159	0.580	0.763	0.310
	GG + GA vs AA	0.462	0.234	0.362	0.782
*UCP3*	GG (15)	94.4 ± 11.9	255.5 ± 52.6	56.9 ± 16.1	51.2 ± 23.2
IVS5-115G/C	GC (29)	98.7 ± 10.2	270.2 ± 52.2	56.5 ± 18.5	43.2 ± 21.5
	CC (6)	100.7 ± 8.6	237.7 ± 54.7	50.0 ± 0.0	48.7 ± 24.6
	GG vs GC + CC	0.159	0.580	0.763	0.310
	GG + GC vs CC	0.462	0.234	0.362	0.782
*UCP3*	TT (15)	94.4 ± 11.9	255.5 ± 52.6	56.9 ± 16.1	51.2 ± 23.2
IVS5-100T/C	TC (29)	98.7 ± 10.2	270.2 ± 52.2	56.5 ± 18.5	43.2 ± 21.5
	CC (6)	100.7 ± 8.6	237.7 ± 54.7	50.0 ± 0.0	48.7 ± 24.6
	TT vs TC + CC	0.159	0.580	0.763	0.310
	TT + TC vs CC	0.462	0.234	0.362	0.782
*UCP3*	II (15)	94.4 ± 11.9	255.5 ± 52.6	56.9 ± 16.1	51.2 ± 23.2
1106delAAG	ID (29)	98.7 ± 10.2	270.2 ± 52.2	56.5 ± 18.5	43.2 ± 21.5
	DD (6)	100.7 ± 8.6	237.7 ± 54.7	50.0 ± 0.0	48.7 ± 24.6
	II vs ID + DD	0.159	0.580	0.763	0.310
	II + ID vs DD	0.462	0.234	0.362	0.782

Data are expressed as the mean ± SD.

*p*-values were calculated by ANOVA. * and bold: *p* < 0.05.

I: insertion, D: deletion, IVS: intervening sequence.

Loci which were not observed polymorphism in Labrador retriever, or were not detected *p*-value are not shown.

Shetland Sheepdogs are considered to have a predisposition to primary hyperlipidemia as determined by the levels of cholesterol, triglycerides, and free fatty acids [28, 29]. Therefore, we investigated the distribution of genotypes for SNPs and indels of the *UCP2* and *UCP3* genes in a population of Shetland Sheepdogs (n = 30). Shiba (n = 30) were also tested as a comparative contrast breed in this study. Statistically significant differences in allele frequency between the two breeds were found in five of the 14 polymorphic sites in *UCP2* (-3629C/G, -2931A/T, -748G/A, -636A/G and IVS6-133delTCTCCCC, Additional file 3). Four SNPs (-4339T/C, -930T/C, 143A/C and IVS3+121T/C) of the 14 *UCP3* polymorphic sites were significantly different in allele frequency between the two breeds (Table 2). Despite the different genetic background in each of the dog breeds [30–32], the different allele frequencies in the *UCP2* and *UCP3* polymorphic site between the two breeds may result from the susceptibility of Shetland Sheepdogs to hypercholesterolemia in a limited number of individuals.

**Table 2:** **Genotyping data and interbreed analysis of DNA polymorphisms in** ***UCP3***

***UCP3*** DNA polymorphism	Genotype	Number of samples				Allele frequency	
		Shiba	Shetland sheepdog	***p***	Allele	Shiba	Shetland sheepdog
-4399C/T	CC	29	30	NS	C	0.98	1.00
	CT	1	0	CC vs CT + TT	T	0.02	0.00
	TT	0	0				
-4339T/C	TT	0	4	p < 0.05	T	0.07	0.37
	TC	4	14	TT + TC vs CC	C	0.93	0.63
	CC	26	12				
-4160G/A	GG	30	30	ND	G	1.00	1.00
	GA	0	0		A	0.00	0.00
	AA	0	0				
-4010C/T	CC	18	13	NS	C	0.75	0.70
	CT	9	16	CC vs CT + TT	T	0.25	0.30
	TT	3	1				
-930T/C	TT	0	4	p < 0.05	T	0.07	0.37
	TC	4	14	TT + TC vs CC	C	0.93	0.63
	CC	26	12				
-803C/T	CC	30	30	ND	C	1.00	1.00
	CT	0	0		T	0.00	0.00
	TT	0	0				
143A/C	AA	20	30	p < 0.05	A	0.82	1.00
	AC	9	0	AA vs AC + CC	C	0.18	0.00
	CC	1	0				
IVS3+26T/C	TT	10	13	NS	T	0.57	0.70
	TC	14	16	TT vs TC + CC	C	0.43	0.30
	CC	6	1				
IVS3+69G/A	GG	18	13	NS	G	0.75	0.70
	GA	9	16	GG vs GA + AA	A	0.25	0.30
	AA	3	1				
IVS3+121T/C	TT	29	14	p < 0.05	T	0.98	0.67
	TC	1	12	TT vs TC + CC	C	0.02	0.33
	CC	0	4				
IVS5-115G/C	GG	10	4	NS	G	0.55	0.37
	GC	13	14	GG + GC vs CC	C	0.45	0.63
	CC	7	12				
IVS5-100T/C	TT	18	13	NS	T	0.75	0.70
	TC	9	16	TT vs TC + CC	C	0.25	0.30
	CC	3	1				
838T/C	TT	28	30	NS	T	0.97	1.00
	TC	2	0	TT vs TC + CC	C	0.03	0.00
	CC	0	0				
1106delAAG	ins ins	18	13	NS	ins	0.75	0.70
	ins del	9	16	II vs ID + DD	del	0.25	0.30
	del del	3	1				

I: insertion, D: deletion. IVS: intervening sequence.

*p*-values were calculated by Fisher’s exact test. *p* < 0.05 NS:not significance. ND: not detection.

The T allele at -4339T/C and -930T/C located in the *UCP3* intron 1 is associated with higher T-Cho levels, as shown by two different experiments: the association between polymorphisms and metabolic data (Table 1), and the distribution of allele of genotype in the breed that is susceptible to hypercholesterolemia (Table 2). These results suggest that the dog *UCP3* gene might be associated with T-Cho levels in a limited number of individuals.

It is known that the peroxisome proliferator activated receptors (PPAR) ligands activate *UCP3* expression [33, 34]. The *UCP3* intron 1 contains that the putative binding elements of MyoG/MyoD, PPARγ/RXRα and SP1/SP3 that enhanced the *UCP3* gene transcription mainly regulated by PPARs in hamster, rat, and mouse [33]. Recently, we find the similar nucleotide sequences of the PPARγ/RXRα element in the dog *UCP3* intron 1 (Canine Genome Draft, NC_006603.3). These findings imply that the dog *UCP3* intron 1 may be associated with regulation of *UCP3* gene expression. Further studies will be needed to demonstrate whether PPAR ligands bind or not this intronic region in dog.

With each genetic study, a different sample size is used to identify the candidate gene associating with genotypes and phenotypes in common diseases (multifactorial diseases) and/or single gene disorders. For instance, genome-wide association studies (GWAS) have reported the candidate gene associated with a mild form of disproportionate dwarfism using 23 cases and 37 controls [35], atopic dermatitis using 91 cases and 88 controls [36], and the chromosomal region of Patellar Luxation using 45 cases and 40 controls [37]. Some of the candidate genes were also tested using more than a hundred samples. The examination of larger sample sizes and further analysis will lead to increased precision of our results. In addition, because the association analysis in this study was performed using only polymorphisms within the *UCP2* and *UCP3* genes, we cannot exclude the possibility that a gene that is closely linked to *UCP3* is causal.

## Conclusions

A genetic association study between polymorphisms in the dog uncoupling protein 2 and 3 genes and metabolic data showed that the SNPs of the *UCP3* intron 1 were associated with T-Cho levels in Labrador Retrievers. Alleles associated with high T-Cho levels of these polymorphisms were also present at higher frequencies in a breed that is susceptible to hypercholesterolemia (Shetland Sheepdogs), than in the control group (Shiba). The results obtained from a limited number of individuals suggest that the *UCP3* gene in dogs may be associated with total cholesterol levels. Therefore, the *UCP3* gene could be an interesting target, not only for lipid metabolism, but also for the treatment and prevention of obesity and metabolic-related diseases in dogs.

## Methods

### Animals and DNA

All animal experiments were approved by The Experimental Animal Ethics Committee in Nippon Veterinary and Life Science University. The blood samples were originally collected at the Veterinary Medical Teaching Hospital at NVLU with the written consent of each owner or the Kyushu Guide Dog Association. The collection of samples was handled by licensed veterinarians only.

Panel 1, for the first SNP discovery, was collected from 11 dogs that represented 11 different breeds: Miniature Dachshund, Welsh Corgi, Labrador Retriever, Shetland Sheepdog, Beagle, Yorkshire Terrier, Dobermann, Whippet, Weimaraner, Papillon, and Shiba. Panel 2 was used for SNP discovery and a study of associations between SNP variants and biochemical parameters; these samples were collected from 50 Labrador Retrievers. Panel 3 was used for SNP discovery and an interbreed analysis was collected from 30 Shetland Sheepdogs and 30 Shibas containing each one animals from Panel 1. A list of breeds and number of individuals are presented in Table 3. Genomic DNA was extracted from whole blood with the Puregene kit (Qiagen, Valencia CA, USA).

**Table 3:** **List of 119 DNA samples from 11 breeds**

DNA samples	Breeds	N	Sex	
			Male	Female
Panel 1^a^	Miniature Dachshund	1		1
	Welsh Corgi	1	1	
	Labrador Retriever	1	1	
	Shetland Sheepdog	1	1	
	Beagle	1		1
	Yorkshire Terrier	1	1	
	Dobermann	1		1
	Whippet	1		1
	Weimaraner	1		1
	Papillon	1		1
	Shiba	1	1	
Panel 2^a,b,d^	Labrador Retriever	50	27	23
Panel 3^a,b,c^	Shetland Sheepdog	30^e^	15	15
	Shiba	30^e^	15	15
Total		119^f^	60	59

N. Number of samples.

a. SNP discovery.

b.SNP genotyping.

c. Interbreed analysis.

d. Association analysis of DNA polymorphisms with biochemical parameters.

e. Include one individual of panel 1.

f. Total numbers of independent individuals.

### PCR

We used sequences of *UCP2* and *UCP3* (Canine Genome Draft, NC_006603.3), to design 15 pairs of primers for amplification of each exon of the *UCP2* and *UCP3* genes (Table 4). Each PCR using TaKaRa Ex *Taq* was performed in a total volume of 25 μl and contained 20 ng genomic DNA, 2.5 μl 10× Ex *Taq* Buffer (including 20 mM Tris–HCl, 100 mM KCl, 0.1 mM EDTA, 1 mM DTT, 0.5% Tween 20, 0.5% Nonidet P-40, 50% Glycerol, 20 mM Mg2^+^), 0.4 mM of each primer, 200 μM dNTP (dATP, dTTP, dCTP and dGTP), and 1*U* TaKaRa Ex *Taq* (TaKaRa, Shiga, Japan). Each PCR using FastStart *Taq* DNA polymerase (Roche, Basel, Switzerland)) was performed in a total volume of 25 μl and contained 20 ng genomic DNA, 2.5 μl 10× reaction Buffer (including 500 mM Tris–HCl, 100 mM KCl, 50 mM (NH_4_)_2_SO_4_, 20 mM MgCl_2_), 0.4 mM of each primer (F12: 0.2 mM of each primer), 200 μM dNTP (dATP, dTTP, dCTP and dGTP), and 1*U* FastStart *Taq* DNA polymerase. If necessary, we used FastStart *Taq* for primer pairs that did not work with TaKaRa Ex *Taq*. The PCR reactions were performed on TaKaRa PCR Thermal Cycler Dice TP600 (TaKaRa). The conditions for PCR are shown in Table 5.

**Table 4:** **Sequences of primers for PCR**

Gene	Fragment	Primer	Primer sequences	Range of PCR amplification ^a^	size	Region ^b^
			(5’-3’)		(bp)	
*UCP2*	F1	UCP2F1-F	CAGCTCTCGGCTTGTGAGC	24304468-24305048	581	Exon 1, Intron 1
		UCP2F1-R	CACAACAGTCAGCAGACTGG			
	F2	UCP2F2-F	CCTTGCTGGAGTGTAATCTG	24305288-24306125	838	Intron 1, Exon 2, Intron 2
		UCP2F2-R	TGGGTTTGCCCAGGTCTTTC			
	F3	UCP2F3-F	TACCAACTCTTCCATACCTC	24307315-24308410	1096	Intron 2, Exon 3
		UCP2F3-R	ATGCAGGCAGCTGTGCCAG			
	F4	UCP2F4-F	TGAGCAGGACAGGACTGTT	24308186-24308944	759	Exon 3, Intron 3, Exon 4, Intron 4
		UCP2F4-R	AAAGGAGCTATACAGCAAATCA			
	F5	UCP2F5-F	TCTCAGAGCATTTACTCTGCT	24309392-24310367	976	Intron 4, Exon 5, Intron 5, Exon 6, Intron 6
		UCP2F5-R	AGAAAAGGCAGTCAGGACTC			
	F6	UCP2F6-F	TCCTCCCCCTCAAACCATCA	24310274-24311183	910	Intron 6, Exon 7, Intron 7, Exon 8
		UCP2F6-R	GAAAGGGAGGTGGTGGGAA			
*UCP3*	F7	UCP3F7-F	ATAGTACTTACCTCATAGGGT	24277647-24278722	1076	5’Fl, Exon 1, Intron 1
		UCP3F7-R	TATCTGTTCTCCATGGCAGC			
	F8	UCP3F8-F	CTAAGGAGCCTTAAGGGAAC	24278114-24278825	712	Exon 1, Intron 1
		UCP3F8-R	TTCAGGGAGAGCTCAGGATC			
	F9	UCP3F9-F	ACGCTACAGGTATGTGTGAG	24281537-24282266	730	Intron 1
		UCP3F9-R	CCTGAAGTGTACAGAGAGCC			
	F10	UCP3F10-F	TAACTAACAGTTTAGGTGAGTC	24282174-24282933	760	Intron 1, Exon 2, Intron 2
		UCP3F10-R	TGCTCAGAGTTCTGTGTGAAG			
	F11	UCP3F11-F	CAGGTCCTTCTGCACCCAG	24283244-24284111	868	Intron 2, Exon 3, Intron 3, Exon 4, Intron 4
		UCP3F11-R	TCATTCTGGGAGTTCCCTCC			
	F12	UCP3F12-F	CCTGTGGCCTTGCAACCAGA	24285138-24285396	259	Intron 4, Exon 5, Intron 5
		UCP3F12-R	TGTTACCTCTGAGTGGTGCC			
	F13	UCP3F13-F	GGCACCACTCAGAGGTAACA	24285377-24286088	712	Intron 5, Exon 6, Intron 6
		UCP3F13-R	TGGGAAGGGATGTTGGATGC			
	F14	UCP3F14-F	GCACTATCGTTACACTCAAGG	24285748-24286088	341	Intron 5, Exon 6, Intron 6
		UCP3F14-R	TGGGAAGGGATGTTGGATGC			
	F15	UCP3F15-F	TAACTGCCTAACACAGAACC	24288288-24289004	717	Intron 6, Exon 7
		UCP3F15-R	TTCAGCCTTTCCTGTACACA			

a. Number of nucleotide position is from canine genome draft (CGD) NC_006603.3.

b. Fl: Flanking region Start codon is located in Exon 3 in *UCP2* and Exon 2 in *UCP3*. Stop codon is located in Exon 8 in *UCP2* and Exon 7 in *UCP3.*

**Table 5:** **Conditions for PCR**

Fragment	Taq ^a^	Initial denature (°C/m) ^b^	Denature (°C/m) ^b^	Annealing (°C/s) ^b^	Extention (°C/m) ^b^	Cycle	Final extention (°C/m) ^b^
F1	F	95/4	95/1	57/30	72/1	35	72/7
F2	E	95/1	95/1	60/30	72/1	35	72/7
F3	F	95/4	95/1	60/10	72/1	30	72/7
F4	E	95/1	95/1	60/30	72/1	35	72/7
F5	F	95/4	95/1	60/30	72/1	35	72/7
F6	E	95/1	95/1	62/30	72/1	35	72/7
F7	E	95/1	95/1	60/15	72/1	34	72/7
F8	F	95/4	95/1	62/30	72/1	35	72/7
F9	F	95/4	95/1	62/30	72/1	35	72/7
F10	E	95/1	95/1	60/30	72/1	35	72/7
F11	F	95/4	95/1	62/15	72/1	32	72/7
F12	E	95/1	95/1	60/30	72/1	35	72/7
F13	E	95/1	95/1	60/30	72/1	35	72/7
F14	E	95/1	95/1	60/30	72/1	35	72/7
F15	E	95/1	95/1	60/30	72/1	35	72/7

a. Taq polymerase: E = ExTaq (TaKaRa), F = Fast start Taq (Roche).

b. m: minutes s: seconds.

### Sequencing and SNP detection

The PCR products were purified with High Pure PCR Product Purification Kit (Roche). Cycle sequencing was then performed with the Big Dye Terminator v3.1 kit (Applied Biosystems, Foster City CA, USA); each reaction was run in a 10 μl reaction volume containing 1 μl purified PCR amplification product, 1 μl Ready Reaction Premix, 1.5 μl 5× Big Dye Sequence Buffer, 1 μl primer (1.6 pmol/μl), and 5.5 μl sterile water. Cycle sequencing reactions were performed with the following conditions: 60 s at 96°C followed by 25 cycles of 10 s at 96°C, 5 s at 50°C and 4 min at 60°C. BigDye Xterminator Purification kits were used according to the manufacturer’s instructions (Applied Biosystems) to purify dye-labeled fragments. Samples were analyzed on an ABI PRISM 310 genetic analyzer (Applied Biosystems). We identified DNA polymorphisms by comparing each sequence with the reference sequence (Canine Genome Draft. NC_006603.3) by BLAST in NCBI (National Center for Biotechnology Information) and GENETYX program Ver. 11(GENETYX Corporation, Tokyo, Japan). The position of identified DNA polymorphism was numbered from the A of the initiator methionine ATG codon as the +1 revealed in exon. In case of intron, a positive number indicates the number of nucleotides away from the previous exon, while a negative number indicates the number of nucleotide away from the next exon.

### Measurement of biochemical parameters

Blood samples were collected into heparinized plastic tubes at least 12 h postprandial. Plasma was separated by centrifugation at 1500× g for 10 min. Glucose (GLU), triglyceride (TG), total cholesterol (T-Cho), and lactate dehydrogenase (LDH) were measured using a Spotchem EM SP-4430 (Arkray, Kyoto, Japan) with the manufacturer’s reagents.

### Statistical analysis

Deviation from the Hardy–Weinberg equilibrium was assessed by the Chi-squared test. SNPAlyze (Dynacom, Chiba, Japan) was used to estimate haplotype frequencies. Genotype frequencies were compared using the Fisher’s exact test. Differences of *p* < 0.05 were considered statistically significant. Associations between genotype frequencies and metabolic data were analyzed by one-way analysis of variance (ANOVA).

## Electronic supplementary material

Additional file 1: **Description and localization of identified DNA polymorphisms in** ***dog UCP2*** **and** ***UCP3*** **genes.** (PDF 19 KB)

Additional file 2: **Association analysis of** ***UCP2*** **DNA polymorphisms with biochemical parameters among healthy Labrador Retrievers.** (PDF 38 KB)

Additional file 3: **Genotyping data and interbreed analysis of DNA polymorphisms in** ***UCP2.*** (PDF 41 KB)
