# Supplementary material for: The genetic association study between polymorphisms in uncoupling protein 2 and uncoupling protein 3 and metabolic data in dogs
Source: BMC Res Notes. 2014 Dec 11;7:904. doi: 10.1186/1756-0500-7-904 (PMC4295406; doi:10.1186/1756-0500-7-904)
Supplement: Supplementary file 1 — Additional file 1: Description and localization of identified DNA polymorphisms in dog UCP2 and UCP3 genes. (PDF 19 KB) [file 13104_2014_3464_MOESM1_ESM.pdf]

Additional file 1. Description and localization of identified DNA polymorphisms in *dog UCP2* and *UCP3* genes.

DNA polymorphisms[a]	Location	Amino Acid	CGD[b]	Nucleotide change[c]
UCP2				
-3629C/G	Intron 1		24304705	TATTTTTCCTCT[C/G]TGTCCTCTGTCT
-3621T/C	Intron 1		24304713	CTCTCTGTCCTC[T/C]GTCTTTTCCTTT
-2951delTTCA	Intron 1		24305383[e]	TTGACTGTTGGC[TTCA/----]TTCAGCTCAGGT
-2931A/T	Intron 1		24305403	GCTCAGGTCATG[A/T]TCTGGGGATGAT
-2913A/G	Intron 1		24305421	GGATGATAGTGG[A/G]ATCCAGCTAGTA
-2613A/C	Exon 2[d]		24305721	CGTGAGACCTTA[A/C]AAAGCCGGGTAA
-916C/T	Intron 2		24307418	TACCTGCCCATC[C/T]CCGTGCCAGACA
-748G/A	Intron 2		24307586	GTACCCGGGATC[G/A]TGTTCCGCTTCG
-636A/G	Intron 2		24307698	AAAAAAAAAATG[A/G]CTTGCGAGAGCT
IVS6-133delTCTCCCC	Intron 6		24310250[e]	TCCCCTCTCCCC[TCTCCCC/-------]CTCCCCCTTTCC
IVS6-108C/T	Intron 6		24310275	CTTTCCGCCTGT[C/T]CTCCCCCTCAAA
IVS7-187insA	Intron 7		24310762-63	GGTATTAAAATA[-/A]GAGACTCACAGG
IVS7-152delA*	Intron 7		24310798	CACAGCTACACA[A/-]CTGTGGAGCTCT
IVS7-106C/T	Intron 7		24310844	CCTTTGTGACAG[C/T]AGCTGGGTTGGA
UCP3				
-4399C/T	Intron 1		24278297	CCTCCCCAGCCG[C/T]GTCAAGAAAGGG
-4339T/C	Intron 1		24278357	CTGTGTCACACC[T/C]CCACACCTGTGT
-4160G/A*	Intron 1		24278536	CCCCTTTTCTGG[G/A]TAGAGGTGGTAC
-4010C/T	Intron 1		24278686	GAACGGGGTCCC[C/T]TGACCCCTGCTT
-930T/C	Intron 1		24281766	CACACTGGGAGA[T/C]GGGGCACCTTCT
-803C/T	Intron 1		24281893	AGGCAGATCAGC[C/T]GCCACTGCCCCC
143A/C	Exon 3	Gln>Pro	24283330	AGGGGGAGAACC[A/C]GGCGACACAGGC
IVS3+26T/C	Intron 3		24283550	ATCCTGTAGACA[T/C]CGAGGGGGTAGG
IVS3+69G/A	Intron 3		24283593	TAATAGATCAGA[G/A]ATGAGGGCAGTA
IVS3+121T/C	Intron 3		24283645	GAAGGGTGGTAA[T/C]AGACATTCGCAA
IVS5-115G/C	Intron 5		24285756	GAAAGCACTATC[G/C]TTACACTCAAGG
IVS5-100T/C	Intron 5		24285771	ACACTCAAGGAG[T/C]CCACAGTTTAGT
838T/C**	Exon 7	Leu>Leu	24288536	ACACCATCCTTT[T/C]TGCGTTTGGGAA
1106delAAG	Exon 7[d]		24288804[e]	GTGCTTTGTCTT[AAG/---]AACACATTTGTT

a. The position of identified DNA polymorphism was numbered from the A of the initiator methionine ATG codon as the +1 revealed in exon. In case of intron, a positive number indicates the number of nucleotides away from the previous exon, while a negative number indicates the number of nucleotide away from the next exon. IVS: intervening sequence

b. CGD: Canine Genome Draft. NC_006603.3 was used as the reference sequence

c. Reference allele given first

d. Untranslated region

e. First nucleotide position of indel sequence

**UCP2* -IVS7-152delA and *UCP3* --4160G/A were identified in panel 2 and others were identified in panel1

**UCP3-838T/C was identified in panel 3
