# Supplementary material for: The genetic association study between polymorphisms in uncoupling protein 2 and uncoupling protein 3 and metabolic data in dogs
Source: BMC Res Notes. 2014 Dec 11;7:904. doi: 10.1186/1756-0500-7-904 (PMC4295406; doi:10.1186/1756-0500-7-904)
Supplement: Supplementary file 2 — Additional file 2: Association analysis of UCP2 DNA polymorphisms with biochemical parameters among healthy Labrador Retrievers. (PDF 38 KB) [file 13104_2014_3464_MOESM2_ESM.pdf]

Additional file 2. Association analysis of *UCP2* DNA polymorphisms with biochemical parameters among healthy Labrador Retrievers.

DNA polymorphism	Genotype	GLU	T-Cho	LDH	TG
UCP2	CC (46)	97.4±10.7	262.7±53.2	56.1±17.2	46.8±22.2
-3629C/G	CG (4)	100.0±10.6	252.0±52.6	52.8±5.5	39.8±24.5
	GG (0)	-	-	-	-
	CC vs CG+GG	0.647	0.701	0.701	0.545
UCP2	DD (46)	97.4±10.7	262.7±53.2	56.1±17.2	46.8±22.2
-2951delTTCA	DI (4)	100.0±10.6	252.0±52.6	52.8±5.5	39.8±24.5
	II (0)	-	-	-	-
	DD vs DI+II	0.647	0.701	0.701	0.545
UCP2	GG (46)	97.4±10.7	262.7±53.2	56.1±17.2	46.8±22.2
-2913A/G	GA (4)	100.0±10.6	252.0±52.6	52.8±5.5	39.8±24.5
	AA (0)	-	-	-	-
	GG vs GA+AA	0.647	0.701	0.701	0.545
UCP2	AA (46)	97.4±10.7	262.7±53.2	56.1±17.2	46.8±22.2
-2613A/C	AC (4)	100.0±10.6	252.0±52.6	52.8±5.5	39.8±24.5
	CC (0)	-	-	-	-
	AA vs AC+CC	0.647	0.701	0.701	0.545
UCP2	TT (46)	97.4±10.7	262.7±53.2	56.1±17.2	46.8±22.2
-916C/T	TC (4)	100.0±10.6	252.0±52.6	52.8±5.5	39.8±24.5
	CC (0)	-	-	-	-
	TT vs TC+TC	0.647	0.701	0.701	0.545
UCP2	II (47)	97.3±10.7	261.6±53.2	56.0±17.0	46.6±22.0
IVS6-133delTCTCCCC	ID (3)	103.7±9.3	266.7±53.5	53.7±6.4	42.0±29.4
	DD (0)	-	-	-	-
	II vs ID+DD	0.315	0.872	0.817	0.734
UCP2	TT (46)	97.4±10.7	262.7±53.2	56.1±17.2	46.8±22.2
IVS6-108C/T	TC (4)	100.0±10.6	252.0±52.6	52.8±5.5	39.8±24.5
	CC (0)	-	-	-	-
	TT vs TC+CC	0.647	0.701	0.701	0.545
UCP2	II (46)	97.4±10.7	262.7±53.2	56.1±17.2	46.8±22.2
IVS7-187insA	ID (4)	100.0±10.6	252.0±52.6	52.8±5.5	39.8±24.5
	DD (0)	-	-	-	-
	II vs ID+DD	0.647	0.701	0.701	0.545
UCP2	II (47)	97.3±10.7	261.6±53.2	56.0±17.0	46.6±22.0
IVS7-152delA	ID (3)	103.7±9.3	266.7±53.5	53.7±6.4	42.0±29.4
	DD (0)	-	-	-	-
	II vs ID+DD	0.315	0.872	0.817	0.734

Data are expressed as the mean ±SD
p -values were calculated by ANOVA. $p<0.05$
I : insertion, D : deletion. IVS: intervening sequence.
Loci which were not observed polymorphism in Labrador retriever, or were not detected *p* -value are not shown.
