# Supplementary material for: The genetic association study between polymorphisms in uncoupling protein 2 and uncoupling protein 3 and metabolic data in dogs
Source: BMC Res Notes. 2014 Dec 11;7:904. doi: 10.1186/1756-0500-7-904 (PMC4295406; doi:10.1186/1756-0500-7-904)
Supplement: Supplementary file 3 — Additional file 3: Genotyping data and interbreed analysis of DNA polymorphisms in UCP2. (PDF 41 KB) [file 13104_2014_3464_MOESM3_ESM.pdf]

Additional file 3. Genotyping data and interbreed analysis of DNA polymorphisms in *UCP2*.

DNA polymorphism	Genotype	Numbers of animals		*p*	Allele	Allele frequency	
		Shiba	Shetland sheepdog			Shiba	Shetland sheepdog
-3629C/G	CC	11	30	p <0.05	C	0.55	1.00
	CG	11	0	CC vs CG+GG	G	0.45	0.00
	GG	8	0				
-3621T/C	TT	30	26	NS	T	1.00	0.93
	TC	0	4	TT vs TC+CC	C	0.00	0.07
	CC	0	0				
-2951delTTCA	ins ins	8	3	NS	ins	0.45	0.37
	ins del	11	16	II+ID vs DD	del	0.55	0.63
	del del	11	11				
-2931A/T	AA	13	30	p<0.05	A	0.65	1.00
	AT	13	0	AA vs AT+TT	T	0.35	0.00
	TT	4	0				
-2913A/G	AA	8	3	NS	A	0.45	0.37
	AG	11	16	AA+AG vs GG	G	0.55	0.63
	GG	11	11				
-2613A/C	AA	11	11	NS	A	0.55	0.63
	AC	11	16	AA vs AC+CC	C	0.45	0.37
	CC	8	3				
-916C/T	CC	8	3	NS	C	0.45	0.37
	CT	11	16	CC+CT vs TT	T	0.55	0.63
	TT	11	11				
-748G/A	GG	30	13	p <0.05	G	1.00	0.70
	GA	0	16	GG vs GA+AA	A	0.00	0.30
	AA	0	1				
-636A/G	AA	30	13	p <0.05	A	1.00	0.70
	AG	0	16	AA vs AG+GG	G	0.00	0.30
	GG	0	1				
IVS6-133delTCTCCCC	ins ins	11	26	p <0.05	ins	0.55	0.93
	ins del	11	4	II vs ID+DD	del	0.45	0.07
	del del	8	0				
IVS6-108C/T	CC	8	3	NS	C	0.45	0.37
	CT	11	16	CC+CT vs TT	T	0.55	0.63
	TT	11	11				
IVS7-187insA	del del	8	3	NS	del	0.45	0.37
	del ins	11	16	DD+DI vs II	ins	0.55	0.63
	ins ins	11	11				
IVS7-152delA	ins ins	30	30		ins	1.00	1.00
	ins del	0	0	ND	del	0.00	0.00
	del del	0	0				
IVS7-106C/T	CC	30	26	NS	C	1.00	0.93
	CT	0	4	CC vs CT+TT	T	0.00	0.07
	TT	0	0				

I : insertion, D : deletion. IVS: intervening sequence.

p -values were calculated by Fisher's exact test. p <0.05 NS:not significance. ND: not detection.
